# Nutri-PEITC Jelly Significantly Improves Progression-Free Survival and Quality of Life in Patients with Advanced Oral and Oropharyngeal Cancer: A Blinded Randomized Placebo-Controlled Trial

**DOI:** 10.3390/ijms24097824

**Published:** 2023-04-25

**Authors:** Aroonwan Lam-Ubol, Jirasak Sukhaboon, Withee Rasio, Peerawitch Tupwongse, Thapana Tangshewinsirikul, Dunyaporn Trachootham

**Affiliations:** 1 Faculty of Dentistry, Srinakharinwirot University, Bangkok 10110, Thailand; aroonwan@gmail.com; 2 Lopburi Cancer Hospital, Lopburi 15000, Thailand; 3 National Cancer Institute, Bangkok 10400, Thailand; 4 Chonburi Cancer Hospital, Chonburi 20000, Thailand; 5 Institute of Nutrition, Mahidol University, Nakhon Pathom 73170, Thailand

**Keywords:** PEITC, Nutri-Jelly, oral cancer, tertiary chemoprevention, p53, clinical trial, survival, quality of life

## Abstract

*TP53* mutation is associated with cancer progression. Novel strategies to reboot p53 are required to stabilize the disease and improve survival. This randomized placebo-controlled trial investigated safety and efficacy of Nutri-PEITC Jelly (a texture-modified nutritious diet fortified with β-phenethyl isothiocyanate (PEITC) on oral cancer. Seventy-two patients with advanced-staged oral or oropharyngeal cancer were randomly assigned to study and control groups, who consumed 200 g of Nutri-Jelly with and without 20 mg of PEITC, respectively, 5 days/week for 12 weeks. Outcomes, including adverse events, health-related quality of life (HRQOL), progression-free survival (PFS), tumor response, serum p53, and cytochrome c, were measured at 0, 1, and 3 months. Results show that the study group had a higher proportion of participants with improved HRQOL, stable disease, and increased serum p53 levels than those in the control group (*p* < 0.001). The PFS time in the study group was significantly longer than that of the control group (*p* < 0.05). Serum cytochrome c levels were non-significantly decreased in the study group. No serious intervention-related adverse events occurred in either group. In conclusion, Nutri-PEITC Jelly intake for 3 months is safe, stabilizes the disease, improves quality of life and progression-free survival, and might re-activate p53 in advanced-stage oral and oropharyngeal cancer patients.

## 1. Introduction

Mutations in the *TP53* gene are robust in cancer and are associated with disease progression [1,2,3]. Novel strategies to target p53 are required to stabilize the disease and improve the survival of advanced-stage cancer patients [4,5]. Compelling pieces of evidence from in vitro and in vivo studies suggest that β-phenethyl isothiocyanate (PEITC), a Brassica vegetable-derived compound may selectively deplete mutant p53 and reactivate p53 function. While the mechanisms were still unclear, PEITC was shown to reactivate p53 by restoring wild-type conformation and function of p53 protein and selectively depleting mutant p53 by direct binding and conformational change [6,7]. PEITC was also shown to retard the growth of several types of cancer, such as breast, oral, liver, and prostate cancer, and leukemia [8,9,10,11,12]. However, it is still unknown if the compound could be effective in retarding cancer progression in patients. To the best of our knowledge, there was only one published case report showing the benefit of PEITC in sensitizing B-cell prolymphocytic leukemia (B-PLL) to salvage R-CHOP (Rituximab, Cyclophosphamide, Hydroxydaunorubicin [doxorubicin hydrochloride], Oncovin [vincristine sulfate], Prednisone or Prednisolone) chemotherapy [13].

Oral and oropharyngeal cancer is the most common type of head and neck cancer, which is among the most common cancers worldwide [14]. Head and neck cancer patients suffer from the disease progression as well as the consequences of the treatments, leading to poor quality of life, diminished treatment response, and poor survival [4,5,6,7,8,9,10,11,12,13,14,15,16]. *TP53* mutation is associated with disease progression, unfavorable treatment response, and poor survival in head and neck cancer patients [17,18,19]. Malnutrition is a common problem for head and neck cancer patients due to the inability to eat and swallow properly [20,21]. To provide the nutritional support and improve the patients’ quality of life, a texture-modified diet with a complete nutrient supply called Nutri-Jelly has been developed by the Dental Innovation Foundation under Royal Patronage, which is a non-profit organization. Our previous clinical trial suggested that consumption of Nutri-Jelly was proven to improve the health-related quality of life of head and neck cancer patients and reduced the necessity of tube feeding [22].

β-phenylethyl isothiocyanate (PEITC) is a phytochemical naturally present in cruciferous vegetables such as watercress, broccoli, wasabi, and cabbage [23,24]. PEITC can inhibit phase I enzymes and induce phase II detoxification enzymes, leading them to the inactivation and excretion of carcinogen metabolites [23,24,25]. Such mechanisms have established PEITC as a promising candidate for primary and secondary prevention of cancer [23]. Furthermore, during the past decade, numerous research studies show promising anti-cancer effects of PEITC. By conjugation with glutathione and other mechanisms, PEITC was selectively toxic to numerous types of cancer cells via reactive oxygen species, (ROS)-mediated mechanisms [26,27,28]. Previous studies by our group and others showed that PEITC selectively killed oral cancer cells with minimal side effects on normal cells [8,9]. Mechanistically, we showed that PEITC led to increased oxidative stress, nuclear translocation of p53 and p21, and cell cycle arrest in *TP53*-mutated oral cancer cells [9]. Consistently, our in vivo study in a *TP53*-mutated oral cancer-xenograft mouse model showed that PEITC at 5 or 10 mg per kg body weight can slow down tumor growth and prolong the survival of cancer-bearing mice along with increased p53 expression in the nucleus of tumor cells in the tissue [9]. These findings suggest that PEITC may reactivate p53 function in oral cancer cells, which could be either restoring wild-type or depleting mutant p53 as previously described in other types of cancer [6,7]. The in vitro and in vivo effects of PEITC against oral cancer cells are promising. However, the effect of PEITC as tertiary chemoprevention in oral cancer patients has never been studied.

To combine the nutritional support benefit with the potential anticancer effect of PEITC, a novel food product called Nutri-PEITC Jelly has been developed by fortification of PEITC into Nutri-Jelly. The Nutri-PEITC Jelly has been tested for its safety by acute, subacute, subchronic, and chronic toxicity tests in animals according to the OECD standardized Guidelines for the Testing of Chemicals (unpublished results). Furthermore, a pharmacokinetic and tolerable study in human volunteers showed that PEITC from Nutri-PEITC Jelly containing 40 mg of PEITC per day can be absorbed rapidly within a few hours and eliminated completely within 24 h [29]. Compared with pure PEITC, Nutri-PEITC Jelly can provide better bioavailability of PEITC evidenced by higher maximum plasma concentration (Cmax) and shorter time to reach maximum plasma level (Tmax) [29,30]. Consecutive repeated doses of Nutri-PEITC Jelly for 5 days demonstrated no accumulation of PEITC [29]. Moreover, our study in healthy volunteers showed that a 5-week continuous intake of Nutri-PEITC Jelly with 20 mg PEITC per day was safe with minimal side effects [31].

Our clinical trial showed a nutrition-improving effect of Nutri-Jelly in head and neck cancer patients. Our animal study showed a promising anti-cancer effect of PEITC against oral cancer. Our clinical trial showed the safety of Nutri-PEITC Jelly in healthy volunteers. However, the effect of Nutri-PEITC Jelly on cancer patients was still unknown. Thus, this study aimed to evaluate the clinical safety and efficacy of Nutri-PEITC Jelly in advanced-stage oral and oropharyngeal cancer patients.

## 2. Results

### 2.1. Participant Flow Chart

**Figure 1:**
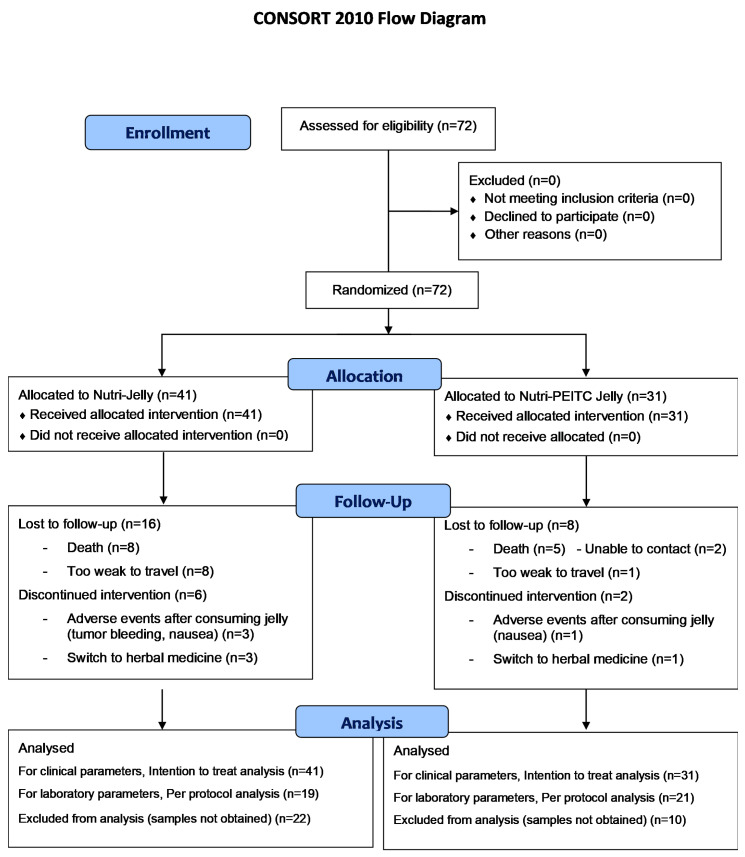
CONSORT participants’ flow diagram. The number of recruited, randomized, and analyzed participants are shown.

As shown in Figure 1, there were 72 eligible participants who were allocated randomly into the study (N = 31) and control (N = 41) groups. In the placebo control group, there were 16 participants who lost follow-up due to death and progressive disease, and 6 participants who discontinued the intervention due to adverse events and switched to herbal medicine treatment. In the Nutri-PEITC Jelly group, there were 8 participants who lost follow-up due to death and progressive disease, and 2 participants who discontinued the intervention due to adverse events or switched to herbal medicine treatment. For clinical parameters, intention-to-treat analysis was used. However, for laboratory parameters, per protocol analysis was applied due to sample unavailability.

### 2.2. Characteristics of Participants

As shown in Table 1, the baseline characteristics of participants in the placebo control group and the study group (Nutri-PEITC Jelly) were comparable. There were no statistically significant differences in gender, occupation, location of primary cancer, disease staging, BMI, age, KPS, and HRQoL scores. Table 2 shows no difference in baseline blood biochemical parameters.

### 2.3. Adverse Events 

As shown in Table 3, no serious intervention-related adverse events occurred in either group. Adverse events reported in both the placebo control and the study groups included nausea, vomiting, burning sensation in the mouth, constipation, dry mouth and throat, and itchy sensation. The study group that received Nutri-PEITC Jelly had a higher percentage of nausea and vomiting and a burning sensation in the mouth, but the percentage of the adverse events was still lower than 30%. Nevertheless, these adverse events may not be entirely related to the interventions as most of them were transient and disappeared without treatment. Diarrhea and tumor bleeding after food consumption was only found in one participant in the placebo control group. After having nausea and vomiting and being unable to eat anything, two participants in the control group and one participant in the study group discontinued the product. As shown in Table 4, the blood chemistry values of both groups were not different throughout the study. Overall, consuming Nutri-PEITC Jelly for 3 months is quite safe in oral and oropharyngeal cancer patients.

### 2.4. Monitoring Plasma PEITC Levels 

**Figure 2:**
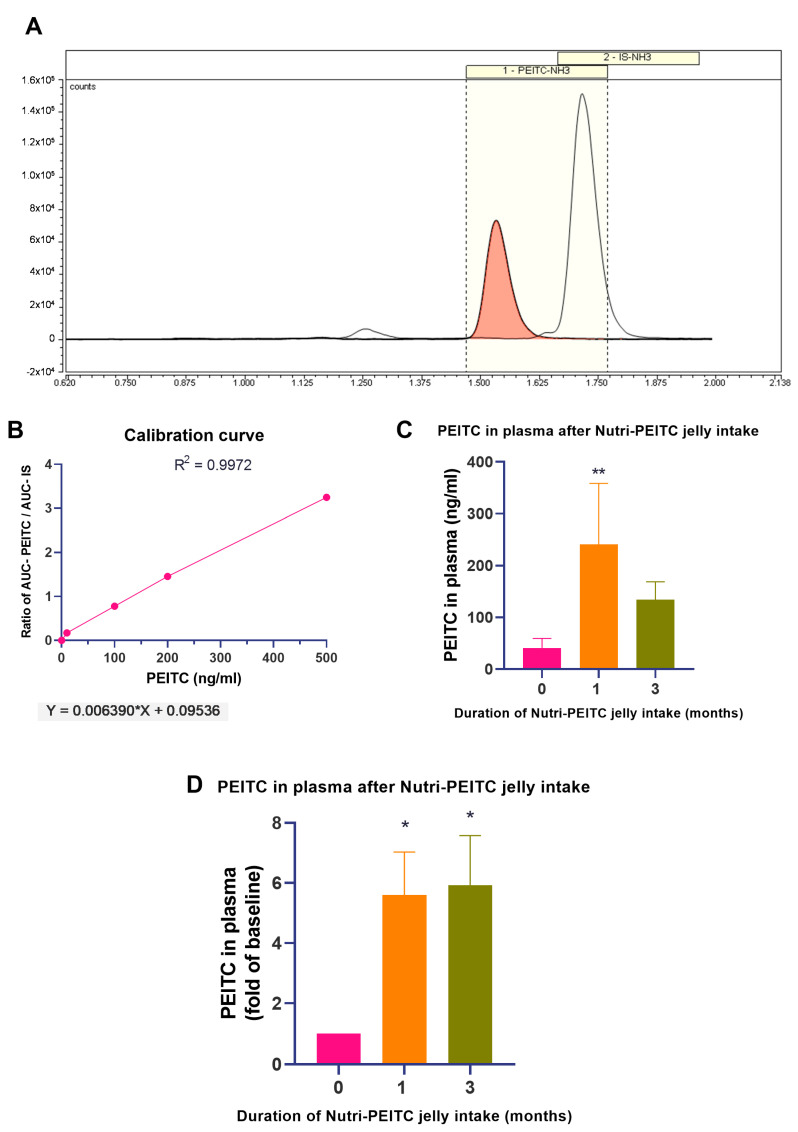
Changes in plasma levels of PEITC after receiving Nutri-PEITC Jelly. (**A**) Representative chromatogram of PEITC and internal standard (IS), (**B**) calibration curve for quantitation of PEITC. Line plots show the ratio between the area under the curve (AUC) of PEITC and the AUC of IS at different concentrations of PEITC (ng/mL), (**C**) comparison of PEITC levels in plasma after Nutri-PEITC Jelly for 0, 1, and 3 months. The bar graph represents mean +/− SEM. ** represents *p* < 0.01, obtained from a Friedman test and Dunn’s multiple comparison test, compared with those of baseline. (**D**) Changes in plasma levels of PEITC after receiving Nutri-PEITC Jelly for 1 and 3 months. The bar graph represents mean +/− SEM of the fold number of PEITC in plasma, compared with individual baseline. * represents *p* < 0.05, a Friedman test and Dunn’s multiple comparison test, compared with those of baseline.

As shown in Figure 2, the plasma PEITC levels in the study group significantly increased up to 6 folds (*p* < 0.05) at 1 and 3 months after the intervention. The data confirm the compliance of Nutri-PEITC intake.

### 2.5. Effects of Nutri-PEITC Jelly on Health-Related Quality of Life (HRQoL)

**Figure 3:**
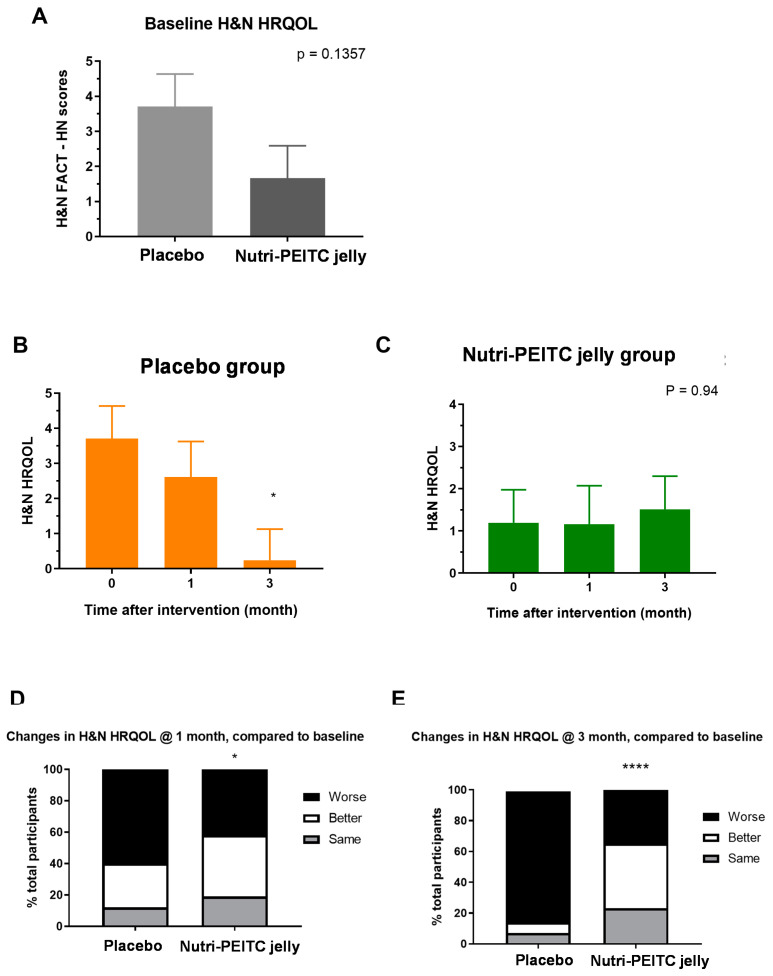
Changes in head and neck health-related quality of life (H&N HRQoL) scores. (**A**) Baseline H&N HRQoL in participants receiving placebo and Nutri-PEITC Jelly as labeled. Bar graph represents mean +/− SEM. *p*-value was from Mann–Whitney Test. (**B**,**C**) Comparison of H&N HRQoL after receiving placebo (**B**) and Nutri-PEITC Jelly (**C**) for 0, 1, and 3 months. Bar graph represents mean+/− SEM. * represents *p* < 0.05, obtained from a Friedman test and Dunn’s multiple comparison test, compared with those of baseline. (**D**,**E**) Changes in H&N HRQoL after receiving placebo and Nutri-PEITC Jelly for 1 (**D**) and 3 (**E**) months. Stacked bars show percentages of participants with worse, better, and the same HRQoL scores when compared with the baseline. * and **** represent *p* < 0.05 and *p* < 0.0001, respectively, compared between groups, obtained from the chi-squared test.

As shown in Figure 3A, at baseline the mean head-and-neck specific HRQoL (H&N HRQOL) score of the study group was lower than those of the control group but the difference was not statistically significant (*p* = 0.1357). As shown in Figure 3B,C, the mean H&N HRQOL of the placebo group had decreased over time, while those in the study group (Nutri-PEITC Jelly) tended to improve. As shown in Figure 3D,E, at 1 and 3 months after the intervention, the study group has a significantly higher proportion of participants with improved head-and-neck specific quality of life (*p* < 0.05 and *p* < 0.0001, respectively).

### 2.6. Effects of Nutri-PEITC Jelly on Tumor Response and Progression-Free Survival Time

**Figure 4:**
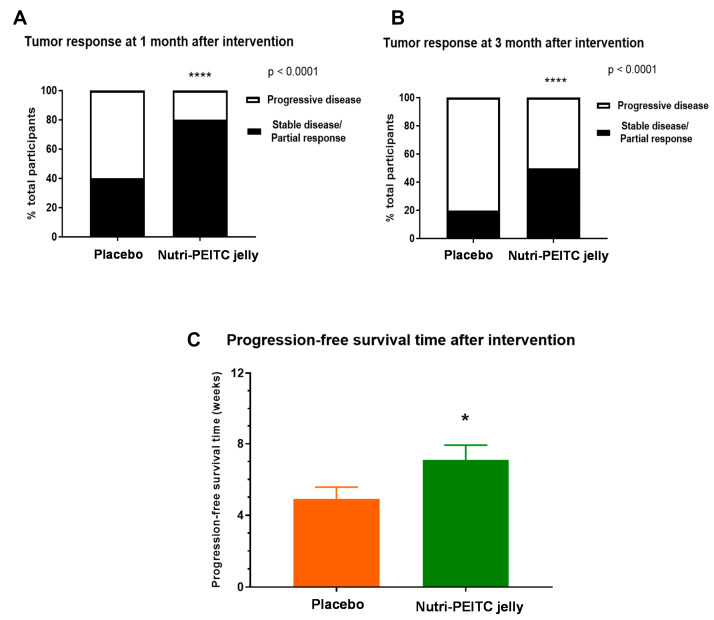
Tumor response and progression-free survival. (**A**,**B**) Tumor response at 1 month (**A**) and 3 months (**B**) after receiving placebo and Nutri-PEITC Jelly as labeled. Stacked bars show percentages of participants with progressive disease (white bar) and stable disease/partial response (black bar) according to RECIST criteria. * and **** represent *p* < 0.05 and *p* < 0.0001, respectively, compared between groups, obtained from the chi-squared test. (**C**) Comparison of progression-free survival between receiving placebo and Nutri-PEITC Jelly as labeled. The bar graph represents mean +/− SEM. * represents *p* < 0.05, obtained from the Mann–Whitney test.

Tumor response was evaluated according to RECIST criteria and shown in Figure 4. As shown in Figure 4A, 1 month after the intervention, the study group had a significantly higher proportion of participants with stable disease (SD) or partial response (PR) than those of the placebo control group (80% vs. 40%, *p* < 0.0001). Consistently, as shown in Figure 4B, 3 months after the intervention, the Nutri-PEITC Jelly group had a significantly higher proportion of participants with SD or PR than those of the placebo control group (50% vs. 20%, *p* < 0.0001). The duration from starting the intervention to developing the progressive disease (PD) was counted as the progression-free survival time. As shown in Figure 4C, the average progression-free survival time in the Nutri-PEITC Jelly group was significantly higher than that of the control group (7.37 ± 4.45 vs. 4.93 ± 4.15 weeks, respectively; *p* < 0.05).

### 2.7. Effects of Nutri-PEITC Jelly on Body Mass Index

**Figure 5:**
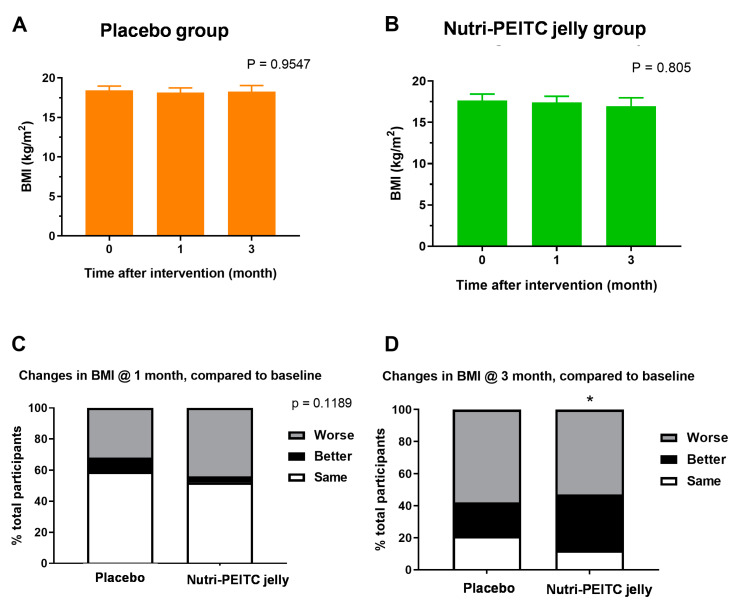
Changes in body mass index (BMI). (**A**,**B**) Comparison of BMI after receiving placebo (**a**) and Nutri-PEITC Jelly (**B**) for 0, 1, and 3 months. The bar graph represents mean +/− SEM. *p*-values were obtained from a Friedman test and compared with those of the baseline. (**C**,**D**) Changes in BMI after receiving placebo and Nutri-PEITC Jelly for 1 (**C**) and 3 (**D**) months. Stacked bars show percentages of participants with worse, better, and the same HRQoL scores when compared with the baseline. * represents *p* < 0.05, respectively, compared between groups, obtained from the chi-squared test.

As shown in Figure 5A,B, there were no significant differences in the mean body mass index after consuming placebo control or Nutri-PEITC Jelly for 1–3 months. However, as shown in Figure 5C,D, 3 months after the intervention, the Nutri-PEITC Jelly group had a significantly higher proportion of participants with improved BMI than that of the placebo control group (40% vs. 20%, *p* < 0.05), while there was no significant difference between groups at 1 month of intervention.

### 2.8. Effects of Nutri-PEITC Jelly on Karnofsky Performance Status Scale (KPS)

**Figure 6:**
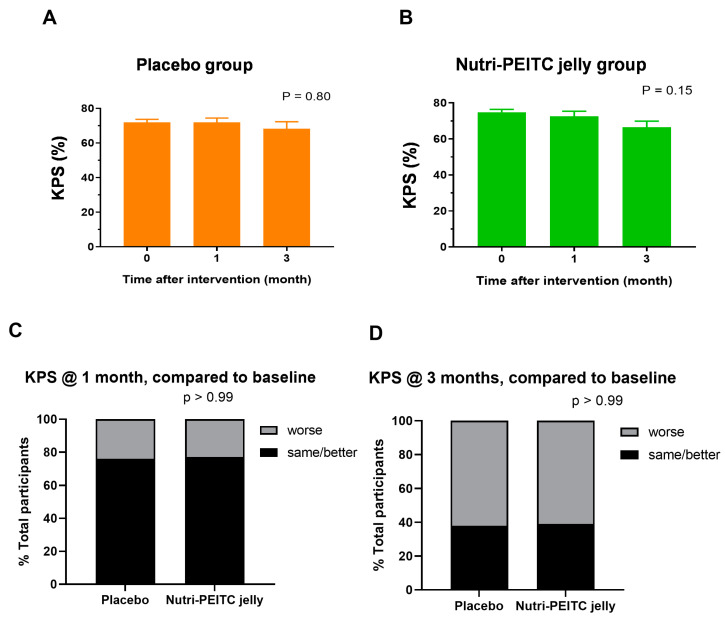
Changes in Karnofsky Performance Status Scale (KPS). (**A**,**B**) Comparison of KPS after receiving placebo (**A**) and Nutri-PEITC Jelly (**B**) for 0, 1, and 3 months. The bar graph represents mean +/− SEM. *p*-values were obtained from a Friedman test and compared with those of the baseline. (**C**,**D**) Changes in KPS after receiving placebo and Nutri-PEITC Jelly for 1 (**C**) and 3 (**D**) months. Stacked bars show percentages of participants with worse, and same/better KPS scores when compared with the baseline. *p*-values were obtained from Fisher’s exact test.

As shown in Figure 6A–D, there were no significant differences in the KPS scores after consuming placebo control or Nutri-PEITC Jelly for 1–3 months.

### 2.9. Effects of Nutri-PEITC Jelly on Serum p53 Levels and Disease Progression

**Figure 7:**
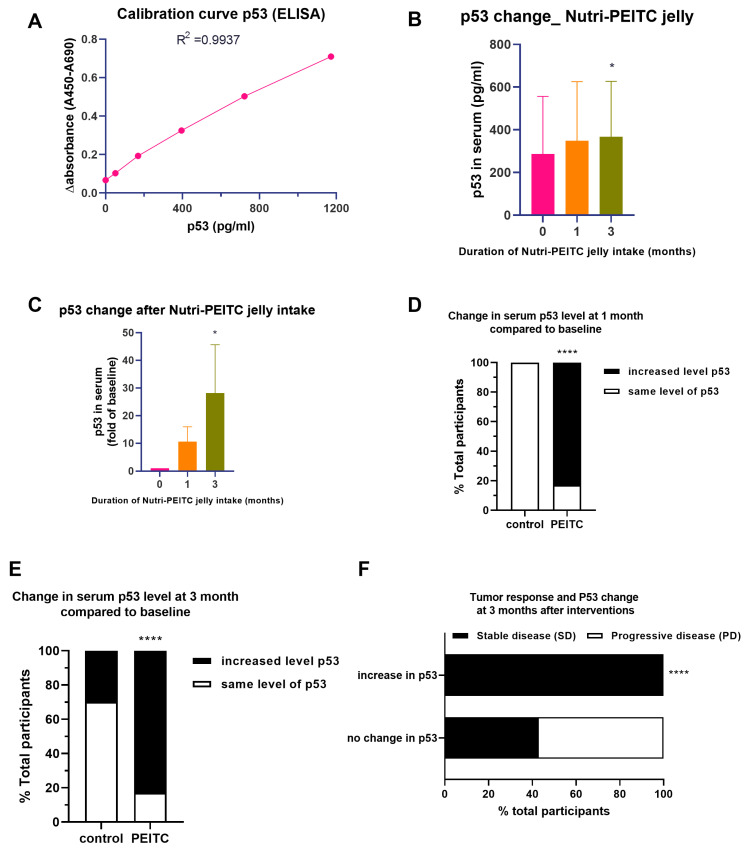
Changes in serum level of p53. (**A**) Calibration curve for quantitation of p53. Line plots show Δ absorbance at 450–690 nm at different concentrations of p53 (pg/mL). R^2^ was obtained from linear regression. (**B**,**C**) Comparison of p53 level in serum (**B**): pg/mL, (**C**) fold number, compared with individual baseline) after receiving Nutri-PEITC Jelly for 0, 1, and 3 months. The bar graph represents mean +/− SEM. * represents *p* < 0.05, obtained from a Friedman test, compared with those of baseline. (**D**,**E**) Changes in serum level of p53 after receiving placebo and Nutri-PEITC Jelly for 1 (**D**) and 3 (**E**) months. Stacked bars show percentages of participants with increased levels of p53 when compared with the baseline. **** represents *p* < 0.0001, respectively, compared between groups, obtained from Fisher’s exact test. (**F**) Tumor response in participants with an increase or no change in serum p53 level. Stacked bars show percentages of stable disease (black bar) and progressive disease (white bar) in participants with increased or no change in serum p53. **** represents *p* < 0.0001, respectively, compared between groups, obtained from Fisher’s exact test.

*TP53* mutation is commonly found in 30–70% of oral and oropharyngeal cancer cases and is associated with poor survival [18]. Restoration of p53 function has been proposed as a new approach to stabilizing cancer progression [32,33]. In this study, we analyzed the serum level of total p53 using ELISA and pan antibodies which could react with both wild-type and mutant p53 protein (Figure 7A). As shown in Figure 7B,C, the average serum p53 levels in the study group were increased after consuming PEITC in a time-dependent manner, with statistical significance seen at 3 months. Compared to the placebo group, the Nutri-PEITC Jelly group had a significantly higher proportion of participants with increased serum p53 protein at 1 and 3 months after intervention (Figure 7D,E; *p* < 0.01 and *p* < 0.001, respectively). Since the increased level of total serum p53 protein could be either a good or bad thing, we further analyzed its association with the SD status. Interestingly, as shown in Figure 7F, at 3 months after intervention all participants with increased p53 levels had SD status, while participants with no change in p53 had either PD or SD status. The significant difference (*p* < 0.0001) suggests that the increased serum p53 level is associated with stable disease tumor status.

### 2.10. Effects of Nutri-PEITC Jelly on Serum Cytochrome c Levels

**Figure 8:**
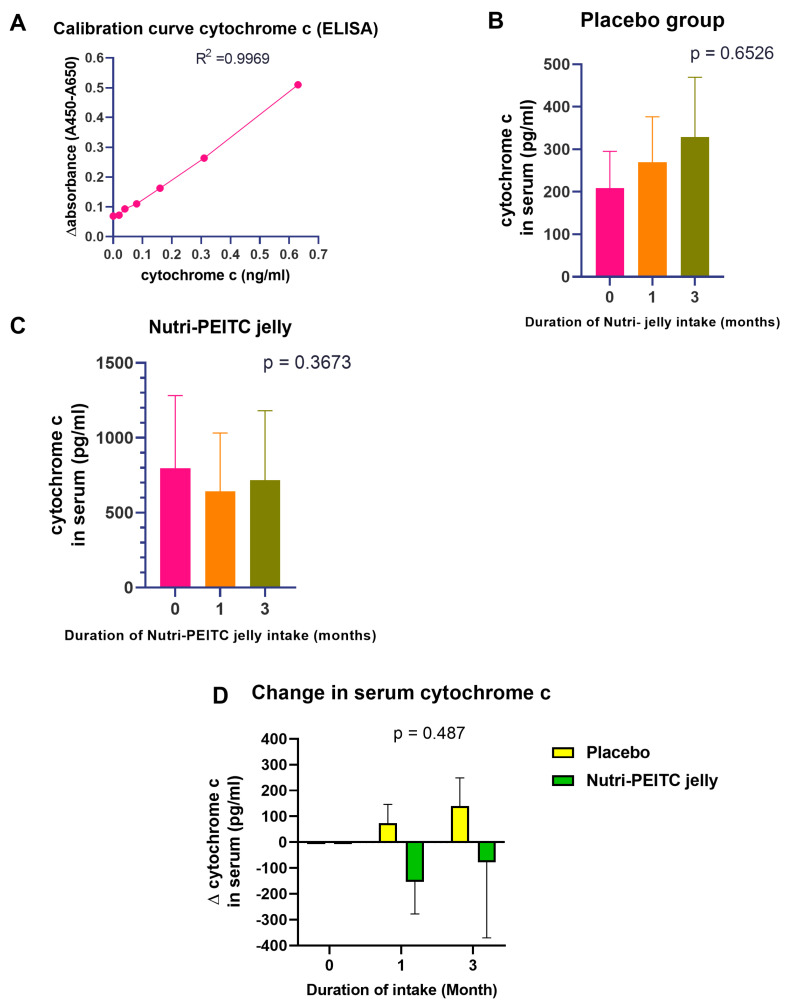
Changes in serum level of cytochrome c. (**A**) Calibration curve for quantitation of cytochrome c. Line plots shows Δ absorbance at 450–690 nm at different concentration of p53 (pg/mL). R^2^ was obtained from linear regression. (**B**,**C**) Comparison of cytochrome c level in serum after receiving placebo (**B**) or Nutri-PEITC Jelly (**C**) for 0, 1, and 3 months. The bar graph represents mean +/− SEM. *p*-values were obtained from a Friedman test and compared with those of the baseline. (**D**) Changes in serum level of cytochrome c after receiving placebo and Nutri-PEITC Jelly for 1 and 3 months. Stacked bars show mean +/− SEM of Δ cytochrome c from individual baseline. *p*-values were obtained from two-way ANOVA.

High serum cytochrome c is associated with poor prognosis and poor survival in cancer patients [34]. In this study, we analyzed the serum level of cytochrome c using ELISA and the specific antibody (Figure 8A). Although the difference between groups was not statistically significant, Figure 8B–D showed that the serum cytochrome c level in the placebo control group tended to increase, while those of the Nutri-PEITC Jelly group tended to decrease.

## 3. Discussion

Head and neck cancer, especially oral and oropharyngeal cancers, are one of the most devastating cancers in its effect on quality of life [35,36]. Problems with chewing and swallowing, taste perception, talking, and facial appearance limit the patient’s physical activities, working capability, and socialization, which eventually lead to malnutrition, low income, and psychological stress [35,36]. Treatment sequelae also severely affect the life quality of the patients [21,37,38]. Despite the advances in treatment modalities, head and neck cancer has had little improvement in survival rate during the past multiple decades [16,39,40]. Therefore, an innovation to stabilize the disease, improve the quality of life, and extend the survival of the patients is urgently needed. In this study, we reported the clinical safety and efficacy of Nutri-PEITC Jelly, an innovative texture-modified diet combining nutritional and anti-cancer properties. The findings suggest that continuous intake of Nutri-PEITC Jelly for 1 and 3 months is tolerable with minimal adverse events and can significantly improve head-and-neck specific quality of life, stable disease (SD) status, and extend the progression-free survival time. Moreover, an investigation of potential anti-cancer mechanisms revealed a significant increase in serum p53 and a non-significant decrease in serum cytochrome c levels in participants taking Nutri-PEITC Jelly.

To our knowledge, only a few human studies have investigated cancer preventive and therapeutic effects of PEITC. A clinical trial in cigarette smokers showed the benefit of PEITC to reduce metabolic activation of lung cancer-specific carcinogens [41]. Our previous clinical trial in meat-eaters showed the efficacy of PEITC-rich vegetable sauce in promoting detoxification and urinary excretion of grilled meat-derived carcinogens [25]. The previous work demonstrates the potential of PEITC in primary cancer prevention. However, the role of PEITC in tertiary prevention in cancer patients has not been reported. Thus, our present study is the first to report the efficacy of functional food containing PEITC in cancer patients. The idea of using functional food for cancer prevention is owing to the paradigm shift from aiming to cure cancer toward reducing mortality and disability by means of cost-effective approaches [42,43]. A recent in vitro study also attempted to identify tertiary chemo-preventive agents [44]. Since oral cancer survival depends on disease progression, nutritional status, and quality of life, Nutri-PEITC Jelly likely has a strong clinical implication in tertiary chemoprevention. In this study, the tumor response was graded according to the RECIST criteria as compared to baseline before the intervention. At baseline, there was no difference in TNM staging between experiment and control groups, suggesting a baseline balance of disease characteristics between the two groups. Compared to the control group, Nutri-PEITC Jelly exhibited a significantly higher percentage of SD/PR status at 1 and 3 months. Nevertheless, when comparing between 1 and 3 months the percentage of SD/PR in the study group still decreased over time. Therefore, Nutri-PEITC Jelly at the dose of 20 mg/day of PEITC did not inhibit the disease progression and the clinical application of this product is rather a functional food for tertiary chemoprevention than a therapy. Future studies with >20 mg/day of PEITC are warranted to investigates its potential therapeutic effect.

In this study, the number of interventions taken was aimed at a nutritional supplement (200 kcal per serving). Supplementation with Nutri-Jelly was previously shown to improve quality of life and reduced the requirement for an NG tube for head and neck cancer patients [22]. In the present study, fortification of PEITC into Nutri-Jelly was shown to better improve the quality of life, which is most likely related to disease stabilization as evidenced by the increased proportion of SD status. The stable disease condition and enhanced quality of life likely contributed to the extended progression-free survival time in patients receiving Nutri-PEITC Jelly. A previous systematic review suggests that cancer-related outcomes were the main factor influencing the quality of life in head and neck cancer patients [45]. Furthermore, previous studies showed that quality of life is a strong predictor of oral cancer survival [46,47].

Reactivation of p53 is a critical approach to regain apoptosis and halt cancer progression [32,33]. In vitro and in vivo studies suggested that PEITC could reactivate p53 function by at least two mechanisms. First, PEITC may selectively deplete mutant p53 by allowing wild-type p53 to regain its activity [8,48]. Second, PEITC could restore wild-type p53 conformation of the mutant p53 [7]. Both mechanisms result in resuming wild-type p53 function in tumor suppression [6,7,8]. Our previous animal study in the oral cancer model showed that PEITC increased total p53 protein expression and nuclear translocation of p53 [9]. This present clinical trial suggested that consuming Nutri-PEITC jelly led to a significant increase in serum p53 levels associated with stable disease status. To our knowledge, this is the first report of an increase in serum p53 associated with favorable outcomes of intervention. Previous observational studies reported elevated serum levels of mutated p53 protein as a marker of *TP53* gene mutation and advanced disease stage [48,49,50,51,52]. According to previous studies in other cancer models, the mechanism of p53 reactivation by PEITC could either resume wild-type p53 function or selectively deplete mutant p53 [6,7]. Unfortunately, in this study we used a p53 pan antibody to measure the total p53 protein (both wild-type and mutant in combination) in serum. Thus, our findings still cannot reveal the actual molecular mechanism. Nevertheless, the observed association between the increase in total p53 protein and stable disease response after consumption of Nutri-PEITC Jelly suggests the possibility that PEITC may favorably reactivate p53 and regain its tumor suppressive function. In fact, a previous study showed that an increase in the p53 protein level might indicate elevated transcriptional activity of *TP53* [53]. Furthermore, a secretome study showed anti-proliferative, pro-apoptotic, and chemosensitivity effects of secreted proteins in media driven by wild-type p53 [54]. Intriguingly, a recent study with a genetically knock-in mice model showed that just partial *TP53* reactivation is adequate to resume tumor suppressive function of p53 resulting in cancer regression [55]. This was quite enthusiastic for head and neck cancer since the *TP53* tumor suppressor gene was the most common somatic genomic alteration in head and neck squamous cells carcinoma (HNSCC) [18]. Although cancer *TP53* status in our participants was unknown, the significant association of increased serum p53 level and stable disease status suggested that anti-cancer effects of PEITC noted in this study likely involved p53. Numerous studies also supported PEITC as a candidate for p53 reactivation [56]. Future studies are warranted to compare responses to treatment in various p53 statuses and identify the exact molecular mechanisms of the increased serum p53 levels. Moreover, a genetic study to investigate p53 alterations in head and neck cancer patients included in the future trial will be beneficial for personalized nutrition.

The strength of this study was the triple-blinded design with matched study and control arms. In addition, we evaluated the participants in various aspects that could affect their quality of life and survival. Subject diaries to record intervention intake as well as serum PEITC levels were analyzed to ensure participants’ compliance. Nevertheless, we encountered some limitations. First, dropout rates were quite high (51.22% and 32.2% in control and study groups, respectively), mostly due to death or progressive disease. Notably, the percentage of dropouts in the control group was higher than that of the study group. The difference may be a result of the intervention’s efficacy in stabilizing the disease in study group [57]. Such high drop-out is commonly seen in clinical trials of late-stage cancer patients, which might affect the reliability of the result [58]. To ensure that our data were reliable, we analyzed all data obtained from the participants by using intention-to-treat analysis for clinical parameters and per protocol analysis for laboratory parameters. We also performed post-hoc power analysis which revealed adequate power based on the sample size obtained: 1.0 for changes in quality of life at 3 months (chi-squared test), 0.917 for tumor response at 1 month (Fisher’s exact test), and 0.843 for progression-free survival (unpaired *t*-test). For future clinical trials in late-stage cancer patients, inclusion criteria shall be modified by recruiting only patients with life expectancy of at least 6 months. Secondly, the genetic background of participants in this study had not been characterized. Thus, the reactivation of p53 as the mechanism is speculation based on our previous in vivo studies and the result of p53 expression change in this study. Third, our study focused on serum p53 levels rather than analyzing p53 levels in tumor samples. Investigating tumor samples could provide more accurate insights into the effects of Nutri-PEITC Jelly on cancer cells and the molecular mechanisms behind its cancer-stabilizing properties. Unfortunately, these patients were biopsied earlier in their disease course in various places in the country. It would be difficult to retrieve the paraffin block of biopsied tissue, and a new tissue biopsy performed during this trial would be too invasive and unjustified. Future studies that include only patients with known genetic background information, i.e., mutated compared to wild-type *TP53* will be very useful in deciphering the p53-mediated mechanism of Nutri-PEITC Jelly. Our previous works have shown the redox-modulating effect of PEITC as a strategy to selectively kill cancer cells in vitro and in vivo [26,27,59,60]. p53 also regulates redox-modulated ferroptosis (lipid peroxidation-induced cell death) [61]. Thus, future mechanistic studies shall focus on p53 reactivation and redox-modulation pathways. Furthermore, there could be other mechanisms behind the cancer-stabilizing mechanism of PEITC, such as epigenetic modulation [62], suppression of inflammatory signaling NF-κB [59,63,64], and inhibition of epithelial-mesenchymal transition as invasion machinery [65]. Future metabolomic studies of clinical specimens from patients receiving Nutri-PEITC Jelly would be worthwhile to characterize the molecular pathways [66].

To address the above-mentioned flaws and limitations, future studies should aim to reduce dropout rates, characterize the genetic background of participants, analyze p53 levels in tumor samples, and explore additional molecular pathways that may contribute to the cancer-stabilizing effects of Nutri-PEITC Jelly.

## 4. Materials and Methods

### 4.1. Ethical Aspects and Setting

Data were collected at Chonburi Cancer Hospital, Lopburi Cancer Hospital, National Cancer Institute, and Maharat Nakhonratchasima Hospital. The study protocol was in compliance with the Declaration of Helsinki and the International Conference on Harmonization Guidelines for Good Clinical Practice (ICH-GCP), and was approved by the Ethical committees for human research of Chonburi cancer hospital (protocol No. 10/2015), Lopburi cancer hospital (Approval No. LEC5712), National cancer institute (protocol No. 302014RC_OUT360), and Maharat Nakhonratchasima hospital (Approval No. 058/2014). This trial was registered with ClinicalTrials.gov (Identifier number: NCT03034603). The protocol can be accessed at https://clinicaltrials.gov/ct2/show/NCT03034603 (accessed on 5 February 2023). All participants provided written informed consent before enrollment.

### 4.2. Study Design, Blinding, Random Allocation, and Concealment 

A multi-center blind randomized placebo-controlled trial was conducted. Seventy-two patients with advanced-stage oral or oropharyngeal cancer were randomly assigned with minimization for age and baseline quality of life scores into the study group (*n* = 31) and control group (*n* = 41). The study and control groups received Nutri-PEITC Jelly and Nutri-Jelly without PEITC (placebo), coded as A and B to conceal their identities. Participants, data collectors, and data analyzers were blinded throughout the study. The placebo jelly and Nutri-PEITC Jelly were packed in the same container with no labels except for the capital letter stating the groups A or B, which were blinded to the investigators, all health personnel and the participants. Furthermore, the researcher who performed randomization, the researcher who provided intervention, the researcher who performed data collection and laboratory analysis, and the statistical analyzer were different people. Standard operating procedure (SOP) was developed by all investigators and used as a standard guide for data collection of all centers.

### 4.3. Participants 

Inclusion criteria of the study population included patients with primary cancers at the lip, oral cavity, oropharynx, pyriform sinus, or hypopharynx (ICD10: C00-C14) whose treatment aimed for palliative care or who denied definitive/standard treatments. If the patients previously received radiotherapy or chemotherapy, those treatments had to be discontinued at least 1 month before enrollment. Cancer had to be diagnosed histopathologically as squamous cell carcinoma and have at least one measurable target lesion. The patients had to have a baseline Karnofsky Performance Status Scale (KPS) of at least 40% or Eastern Cooperative Oncology Group (ECOG) of 0–3, and have acceptable laboratory values (white blood cells ≥ 1.5 × 10^9^ cells/L (≥1500 cells/mm^3^), platelets ≥ 100 × 10^9^/L; Hb ≥ 9.0 g/dL), renal function (creatinine clearance using the CKD-EPI formula (Chronic Kidney Disease Epidemiology group) ≥ 50 mL/min) and hepatic function (SGOT, SGPT, serum bilirubin not more than 1.5 times of upper normal limits). Moreover, participants had to be able to consume the intervention either by mouth or tube feeding for 3 months without aspiration and able to communicate well. Exclusion criteria included patients who received N-acetylcysteine within 3 days before enrollment or continuously, ongoing dialysis, were pregnant or breastfeeding, had uncontrolled systemic diseases or infection, and had a high risk of aspiration. The participants were discontinued from the study if they demonstrated serious adverse events or stopped taking the intervention for more than 2 weeks. All participants signed written informed consent before enrollment into the study and could leave the study whenever they wished.

### 4.4. Sample Size

The estimated sample size was identified by a priori power analysis using G Power 3.1. The effect size was calculated from our previous study on head and neck cancer patients receiving palliative radiotherapy [22] by using quality of life as the primary outcome. Based on the calculation, at least 30 participants per group were required to achieve 90% power at α = 0.05 significance level. Accounting for possible drop-out, no more than a total of 96 participants were expected. Data were collected for three years. Finally, completed data were from 72 patients composed of 31 patients in the study group and 41 patients in the control group. We recruited more subjects in the control than the study group to obtain enough samples for laboratory analyses on account of its higher drop-out rate. Post-hoc power analysis revealed a power of 1.0 for changes in quality of life at 3 months (chi-squared test), 0.917 for tumor response at 1 month (Fisher’s exact test), and 0.843 for progression-free survival (unpaired *t*-test), which are considered adequate.

### 4.5. Interventions

**Figure 9:**
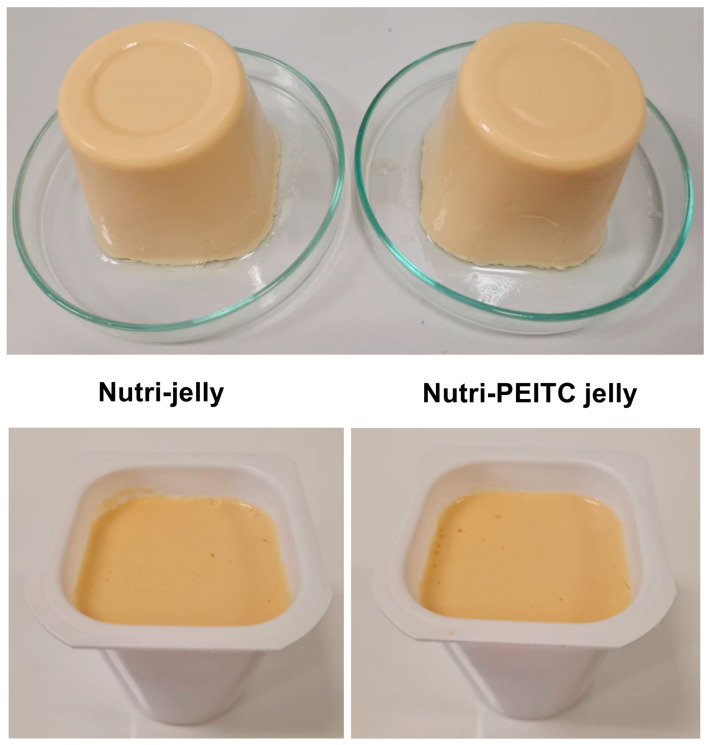
Nutri-Jelly and Nutri-PEITC Jelly: the similar appearance of Nutri-Jelly (left panel) and Nutri-PEITC Jelly (right panel). The bottom panel shows the 100 g cup products.

Nutri-Jelly and Nutri-PEITC Jelly products (Figure 9) were provided by the Dental Innovation Foundation under Royal Patronage. The products were prepared according to the regulation of the Thai Food and Drug Administration (FDA) and HALAL. The products were manufactured in ultra-high temperature (UHT) processing and aseptic filling system under international standards (Good Manufacturing Practice (GMP), Hazard Analysis and Critical Control Points (HACCP), and International Organization for Standardization (ISO) 22000). The products also passed microbiological tests from outside certified laboratories according to the regulation of food in hermetically sealed containers. Nutri-Jelly Mango Flavored is a Thai FDA-registered product with No. 10-1-09760-5-0002. The product is lactose-free. Nutri-PEITC Jelly is the PEITC-fortified version of Nutri-Jelly. Nutri-Jelly and Nutri-PEITC Jelly were both made from similar ingredients (water, mango puree, milk powder, sugar, whey protein, rice bran oil, INS420(i), INS 330, INS 428, INS 406, INS 415) except food-grade PEITC (C6H5CH2CH2NCS, Sigma-Aldrich, St. Louis, MO, USA) was added in Nutri-PEITC Jelly. Each cup of 100 g Nutri-PEITC Jelly contained about 10 mg PEITC (0.01% weight/weight (*w/w*). For quality control of different batches, about 1% of the product was sampled for measurement of PEITC by LC/MS-MS [25]. The acceptable range of PEITC for each batch is 0.006–0.012% PEITC *w*/*w* with the intra-batch coefficient of variation (CV) of <15%. The products were stored at room temperature. The nutrition values of Nutri-Jelly and Nutri-PEITC Jelly were shown in Table 5. The participants in the control and study groups were asked to consume two cups of Nutri-Jelly or Nutri-PEITC Jelly, respectively. Thus, the study group received 20 mg of PEITC per day.

### 4.6. Study Procedure

Baseline assessment was performed before the randomization, including physical examination, cancer size, location and stage, complete blood count, and liver and kidney function tests. General information about the participants, including age, sex, height, and medical history was retrieved from patients’ charts. The participants were asked to consume either 2 cups of 100 g (total of 200 g) of Nutri-Jelly or Nutri-PEITC Jelly in the morning on Monday to Friday (5 days a week) for 3 months. All participants were asked to record their use of the product daily in subject diaries to ensure adherence to the intervention protocol. The intake of paracetamol was also recorded. The outcome measures were evaluated at 0, 1, and 3 months after interventions. The primary outcome measure included adverse events, health-related quality of life (HRQoL), nutritional status, progression-free survival (PFS), and tumor response. The secondary outcome measures were functional assessment outcome (Karnofsky performance status scale: KPS) and serum p53 and cytochrome c levels.

Questionnaires, body weight measurements, blood collection, and tumor size measurement by caliper were performed at all time points. CT scan was performed at 0 and 3 months.

### 4.7. Outcomes

#### 4.7.1. Adverse Events

The physicians assessed the adverse events at 1 and 3 months after the intervention by physical examination, blood hematology, and chemistry. Moreover, adverse events such as nausea, vomiting, and gastrointestinal disturbance were recorded and reported throughout the study period by the participants. The adverse events were graded by the physicians for severity, seriousness, and relatedness to the intervention.

#### 4.7.2. Health-Related Quality of Life and Karnofsky Performance STATUS Scale

Health-related quality of life (HRQoL) was determined by using the Thai version of the FACT-HN questionnaire form after receiving approval from FACIT [67,68,69,70]. The participants were evaluated in several domains including physical, social and family, emotional and functional well-being, and head and neck-related symptoms by completing the questionnaire or interviewing. The higher scores represented better HRQoL. Karnofsky performance status scale for functional impairment was evaluated by a physician using standard criteria [68,71]. Higher scores indicated better performance.

#### 4.7.3. Nutritional Status

Nutritional status was evaluated by body mass index and the levels of serum albumin. Body weight was measured by using a body composition monitor machine (TANITA BC-730, Tanita Corporation, Tokyo, Japan). Body mass index (kg/m^2^) was calculated from body weight/height^2^.

#### 4.7.4. Tumor Responses and Progression-Free Survival Time

Progression-free survival (PFS) time was calculated from the time of the intervention until any signs or symptoms of progressive disease be recorded [69,72]. According to the National Cancer Institute, USA, tumor response is defined as a standard way to measure how well a cancer patient responds to treatment. It is based on whether tumors shrink, stay the same, or get bigger [73]. Tumor responses were investigated according to the response evaluation criteria in solid tumors (RECIST) criteria version 1.1 [73], by using a caliber or CT scan, when applicable, of the target lesion. Complete response (CR) was recorded if all target lesions disappeared after intervention. Partial response (PR) was recorded when there was a 30% decrease in the sum diameter of target lesions after intervention. Progressive disease (PD) was recorded when there was a 20%, and at least 5 mm, increase in the sum diameter of the target lesions after intervention. Stable disease (SD) was recorded when the changes were not sufficient to qualify CR, PR, or PD [73]. Therefore, the SD implies that the disease is subsided with no significant progression [74]. To measure tumor response, the baseline size of tumor mass (target lesion) was measured by using a Vernier caliper and computer tomography (CT) scan at 0 months before intervention. Then, at 1 month the tumor size was measured again using Vernier caliper. At 3 months the tumor size was re-measured by using a Vernier caliper and computer tomography (CT) scan. The tumor sizes at 1 and 3 months were compared with those of the baseline of each participant. Then, tumor response as CR, PR, SD, or PD was concluded according to the RECIST criteria.

#### 4.7.5. Serum Levels of p53 and Cytochrome c

The serum was centrifuged at 4 °C for 10 min at 1000× *g* (3000 rpm). Then, the serum was aliquot and stored at −80 °C until ready to be tested. The p53 ELISA assay was performed by using a p53 pan ELISA kit (Catalog No. 11 828 789 001, Roche, Basel, Switzerland). The assay was designed to recognize a conserved, pantropic, denaturation-stable antigenic determinant of the p53 protein. Briefly, 100 µL of serum samples were plated in duplicate in a 96-well microtiter plate. The plate was pre-coated with biotin-labeled anti-p53 capture antibodies. Then 100 µL of anti-p53-pan-peroxidase detection antibody was added to all wells. The plate was then incubated at room temperature on a shaker for 2 h. After the washing step, tetramethylbenzidine (TMB) substrate was added, followed by a stop solution. The assay results were quantitated spectrophotometrically at 450 nm (reference wavelength 690 nm) using a microplate reader. The calibration curve was generated and used for the calculation of p53 concentration (pg/mL).

The Cytochrome c ELISA assay was performed by using a Cytochrome c Human ELISA Kit (Catalog No. ab119521, Abcam, Cambridge, UK). Briefly, 100 µL of serum samples were plated in duplicate in a 96-well microtiter plate pre-coated with anti-Cytochrome c monoclonal antibody. Then 50 µL of biotinylated detection antibody was added to each well. The plate was then incubated at room temperature for 2 h. After the washing step, 100 uL of streptavidin-horseradish peroxidase was added and incubated at room temperature for 1 h. The plate was then washed, followed by the addition of TMB substrate and stop solution. Optical density was measured by spectrophotometer at 450 nm (reference wavelength 620 nm) using a microplate reader.

#### 4.7.6. Plasma Level of PEITC

To ensure that the participants did consume Nutri-PEITC Jelly, the plasma level of PEITC was measured by using Liquid chromatography Tandem Mass Spectrometry (LC-MS/MS), as previously described [25,75]. A standard curve was generated by using control plasma samples spiked with multiple levels of PEITC concentration (0–500 ng/mL). 3-phenyl propyl isothiocyanate (PPITC) was used as an internal standard (IS) [76]. Both PEITC and PPITC were purchased from Sigma–Aldrich. Extraction of PEITC was performed by adding 600 µL of hexane using a positive displacement pipette to 250 µL of plasma standard or sample and mixing for 30 s with a high-velocity vortex mixer. Then, the mixture was centrifuged at 1000× *g*, 25 °C for 3 min and 500 µL of the top hexane layers was collected into a 2 mL tube. The hexane extraction was performed twice and the supernatant from both times was combined. Then, 400 µL of 2 M ammonia in methanol was added to the hexane extract for ammonia derivatization. The samples were vortexed for 30 s and incubated with shaking on a microplate shaker (Fisher Scientific, Hampton, NH, USA) at room temperature for 4 h. After that, drying in a speed vacuum evaporator (CentriVap Benchtop Vacuum Concentrator, Labconco, Kansas City, MO, USA) at 55 °C for 40 min was performed to remove solvents. Then, 250 µL of acetonitrile water in a ratio of 3:2 was added to reconstitute, followed by mixing with vortex for 1 min. All samples were filtered through a 0.2 µm nylon filter and stored at −20 °C before analysis. The ammonia-derivatized PEITC and PPITC (IS) were measured by an ultra-high performance liquid chromatography-tandem mass spectrometry (LC-MS/MS) system, comprising a UHPLC model Ultimate 3000 (Thermo Scientific, Waltham, MA, USA) and a TSQ Quantis Triple Quadrupole Mass spectrometer (Thermo Scientific, Waltham, MA, USA). LC condition includes injection of 5 µL each sample and run through a Hypersil GOLD™ C18 column 100 × 2.1 mm, particle size 1.9 μm with a mobile phase of acetonitrile: 5 mM formic acid (50:50) at an isocratic flow rate of 0.3 mL/min and a run time of 3.5 min. MS condition includes positive ion electrospray ionization (+ESI) with spraying voltage at 3500 V, sheath gas of 50 arbitrary units (Arb), auxiliary gas of 10.0 Arb, ion transfer tube (ITT) of 325 °C, and vaporizing temperatures of 350 °C, respectively. The retention time of PEITC-NH3 (phenethyl thiourea) was 1.6 min with the mass-to-charge ratio (*m*/*z*) of precursor, quantified, and confirmed product ions at 181, 105.13, and 77.05, respectively. The collision energy of 18.18 and 38.82 V was used for the transition of quantified and confirmed masses, respectively. The retention time of IS-NH3 was 1.7 min with the mass-to-charge ratio (*m*/*z)* of precursor, quantified, and confirmed product ions at 195, 91, and 136, respectively. The collision energy of 26 and 14 V was used for the transition of quantified and confirmed masses, respectively. RF lenses of 129 V and 148 V were used for PEITC-NH3 and IS-NH3, respectively.

### 4.8. Statistical Analysis

The primary efficacy parameters were analyzed for the intention-to-treat (ITT) using the last observation carried forward (LOCF) method for the replacement of missing data points.

The normality of data distribution was verified by a D’Agostino and Pearson omnibus test. Parametric statistical tests were used only when the data passed the normality test (*p* > 0.05). Comparison of baseline characteristics in study and control groups was analyzed by independent *t*-test, Mann–Whitney test, chi-squared, or Fisher’s exact test, as appropriate. The concentrations of plasma PEITC, serum p53, and cytochrome c were calculated by using linear regression of the calibration curve. Comparison of data among different time points was performed by using two-way ANOVA, followed by the Bonferroni test for multiple comparisons. A *p*-value < 0.05 was considered statistically significant. Biochemical data, which contained some missing values, were analyzed by using mixed-effects model (time × group) with the Geisser greenhouse correction. In the end, post-hoc power analysis was performed to ensure an adequate sample size to gain at least 90% power. Sample size and power were calculated by G Power 3.1. Graphing and statistical analysis were performed by GraphPad Prism 5.0.

## 5. Conclusions

The findings of this study suggest that intake of Nutri-PEITC Jelly with 20 mg PEITC/day for 1–3 months is safe with minimal adverse effects. The supplementation may re-activate p53, stabilize the disease, improve the quality of life and progression-free survival in patients with advanced-stage oral and oropharyngeal cancer. Further studies in a larger population and various dosages are warranted to confirm that Nutri-PEITC Jelly could be functional food for tertiary chemoprevention in oral and oropharyngeal cancer.

## Figures and Tables

**Table 1:** Baseline characteristics.

Characteristics	Control Group (N = 41)	Study Group (N = 31)	
	N (%)	N (%)	*p*-Value
**Gender** - Male - Female	24 (59) 17 (41)	25 (81) 6 (19)	0.07 ^†^
**Occupation** - Agriculture - Employee - Unemployed	14 (34) 16 (39) 11 (27)	11(35) 12 (39) 8 (26)	0.99 ^‡^
**Location of primary cancer** - Oral cavity - Tonsil and oropharynx - Pyriform sinus	24 (59) 11 (27) 6 (15)	18 (58) 7 (23) 6 (19)	0.83 ^‡^
**Staging T (tumor size)** - T1–T2 - T3–T4	14 (34) 27 (66)	9 (29) 22 (71)	0.79 ^†^
**Staging N (lymph node involvement)** - N0–N1 - N2–N3	20 (49) 21 (51)	16 (52) 15 (48)	>0.99 ^†^
**Staging M (distant metastasis)** - M0 - M1	40 (98) 1 (2)	31 (100) 0 (0)	>0.99 ^†^
	**mean ± SD**	**mean ± SD**	***p*-value**
**BMI (kg/m^2^)**	18.3 ± 3.3	17.2 ± 3.6	0.15 ^§^
**Age (years)**	63.3 ± 13.8	62.5 ± 12.1	0.67 ^§^
**KPS score (%)**	69.5 ± 10.5	72.6 ± 10.3	0.22 ^§^
**HRQoL**	3.7 ± 5.9	1.2 ± 4.3	0.14 ^§^

*p*-values were from ^†^ Fisher’s exact test, ^‡^ chi-squared test, and ^§^ Mann–Whitney test.

**Table 2:** Baseline blood biochemical parameters.

Characteristics	Control Group (N = 41)	Study Group (N = 31)	*p*-Value
**Hemoglobin (gm/dL)**	11 ± 1.5	10.4 ± 1.4	0.09 ^†^
**Hematocrit (%)**	33.5 ± 4.3	31.8 ± 4.0	0.08 ^†^
**WBC count (cells/mm^3^)**	8737 ± 3364	10,035 ± 5082	0.73 ^‡^
**RBC (cells/mm^3^)**	4114 ± 669.1	3959 ± 774.3	0.34 ^‡^
**MCV (fL)**	82.3 ± 10.7	81.1 ± 9.6	0.37 ^‡^
**MCH (pg)**	27 ± 3.7	26.7 ± 3.2	0.43 ^‡^
**MCHC (g/dL)**	32.8 ± 1.0	32.9 ± 1.0	0.63 ^‡^
**Platelets (cells/mm^3^)**	325,863 ± 136,124	357,355 ± 162,560	0.59 ^‡^
**BUN (mg/dL)**	12.8 ± 4.8	12.8 ± 4.9	0.94 ^†^
**Creatinine (mg/dL)**	0.7 ± 0.2	0.8 ± 0.2	0.09 ^‡^
**Cholesterol (mg/dL)**	165.4 ± 42.3	174.2 ± 60.6	0.65 ^†^
**LDL (mg/dL)**	100.2 ± 37	102.9 ± 49	0.87 ^†^
**HDL (mg/dL)**	45.2 ± 14.4	48.4 ± 10.1	0.27 ^‡^
**SGOT (U/L)**	22.3 ± 8.8	24.7 ± 9.9	0.24 ^‡^
**SGPT (U/L)**	15.8 ± 11.4	17.6 ± 12.8	0.39 ^‡^
**Bilirubin (mg/dL)**	0.4 ± 0.2	0.4 ± 0.2	0.55 ^‡^

This table shows mean ± SD of each characteristic as specified. *p*-values were obtained from ^†^ unpaired *t*-test and ^‡^ Mann–Whitney test.

**Table 3:** Adverse events.

Adverse Events	Control Group (N = 41)	Study Group (N = 31)	Details
	N (%)	N (%)	
Nausea and vomiting	4 (9.8)	7 (22.6)	Two participants in the control group and one participant in the study group discontinued the product, the rest had transient symptoms that disappeared without treatment
Burning sensation in the mouth	4 (9.8)	5 (16.1)	Transient symptoms that disappeared without treatment
Constipation	4 (9.8)	2 (6.5)	One participant in the study group received laxatives for 3 days. The rest had transient symptoms that disappeared without treatment
Dry mouth and throat	3 (7.3)	3 (9.7)	Transient, the symptom disappeared without treatment
Itchy sensation in the mouth	1 (2.4)	1 (3.2)	Transient symptoms that disappeared without treatment
Diarrhea	1 (2.4)	0	Transient symptoms that disappeared without treatment
Tumor bleeding after food consumption	1	0	One participant in the control group discontinued the product

The table shows the number of participants with the specified adverse events. Percent of the participant from total participants was shown in parentheses.

**Table 4:** Changes in blood biochemical parameters.

	Baseline		1 Month Follow-Up		3 Months Follow-Up		*p*-Value ^†^
Parameters	Control Group	Study Group	Control Group	Study Group	Control Group	Study Group	
**Hemoglobin (gm/dL)**	11 ± 1.5	10.4 ± 1.4	10.9 ± 1.5	10.4 ± 1.5	10.5 ± 1.2	9.8 ± 1.7	0.96
**Hematocrit (%)**	33.5 ± 4.3	31.8 ± 4.0	31.7 ± 4.7	33.3 ± 4.5	32.2 ± 3.6	29.8 ± 5.1	0.75
**WBC count (×10^3^ cells/mm^3^)**	8.7 ± 3.4	10.0 ± 5.1	9.8 ± 6.1	9.8 ± 3.6	9.8 ± 5.0	10.0 ± 4.0	0.68
**RBC (10^3^ cells/mm^3^)**	4.1 ± 0.7	3.9 ± 0.8	4.0 ± 0.7	4.0 ± 0.7	3.9 ± 0.5	3.8 ± 0.8	0.87
**Platelets ×10^5^cells/mm^3^)**	3.2 ± 1.4	3.5 ± 1.6	3.5 ± 1.4	3.3 ± 1.4	3.5 ± 1.4	3.5 ± 1.7	0.88
**BUN (mg/dL)**	12.8 ± 4.9	12.8 ± 4.7	13.7 ± 4.5	14.9 ± 9.2	12.6 ± 6.1	13.4 ± 5.5	0.63
**Creatinine (mg/dL)**	0.8 ± 0.2	0.8 ± 0.2	0.7 ± 0.2	0.9 ± 0.3	0.8 ± 0.4	0.9 ± 0.2	0.36
**Cholesterol (mg/dL)**	165.4 ± 41.7	174.2 ± 59.1	162.4 ± 40.6	171.6 ± 41.7	165.7 ± 51.0	157.1 ± 31.2	0.09
**SGOT (U/L)**	22.3 ± 8.8	24.7 ± 9.8	22.25 ± 9.1	25.6 ± 9.9	23.0 ± 11.5	26.9 ± 14.8	0.96
**SGPT (U/L)**	15.8 ± 11.4	17.6 ± 12.7	15.7 ± 12.7	18.1 ± 8.8	18.8 ± 18.8	18.8 ± 15.1	0.98
**Bilirubin (mg/dL)**	0.4 ± 0.2	0.4 ± 0.2	0.5 ± 0.2	0.6 ± 0.6	0.5 ± 0.3	0.5 ± 0.3	0.82

This table shows the mean ± SD of each characteristic as specified. *p*-values were obtained from ^†^ Mixed-effects model (time × group).

**Table 5:** Nutrition values of Nutri-Jelly and Nutri-PEITC Jelly per 100 g cup.

	Nutri-Jelly	Nutri-PEITC Jelly
Total energy (kcal)	130	110
Energy from fat (kcal)	35	30
Total fat (g)	4	3
Saturated fat (g)	2	2
Cholesterol (mg)	15	15
Protein (g)	5	5
Total carbohydrate (g)	18	16
Dietary fiber (g)	2	<1
Sugar (g)	12	9
Sodium (mg)	45	45
Vitamin A (μg RE)	64	64
Vitamin B1 (mg)	0	0
Vitamin B2 (mg)	0.17	0.34
Calcium (mg)	120	120
Iron (mg)	0.3	1.5

## Data Availability

All data collected in this project have been described in this work. No further data are available. Since the participants only gave consent to report the summary of data, no individual data can be shared.
